# Common hepatic duct stenosis secondary to an absorbable ligation clip following laparoscopic cholecystectomy: A case report

**DOI:** 10.1097/MD.0000000000049069

**Published:** 2026-05-29

**Authors:** Kun He, Mengyi Xie, Xiaojin Gao, Houzu Zhou, Wei Cui, Yao Liu, Jingdong Li

**Affiliations:** a Department of Hepatobiliary Surgery, Jintang Second People’s Hospital, Chengdu, Sichuan, China; b Sichuan Branch of National Clinical Research Center for Digestive Diseases, Affiliated Hospital of North Sichuan Medical College, Nanchong, Sichuan, China; c Department of Hepatobiliary Surgery, Zigong Fourth People’s Hospital, Zigong, Sichuan, China.

**Keywords:** absorbable ligation clip, cholecystitis, common hepatic duct stenosis, cystic duct variations, gallstones, laparoscopic cholecystectomy

## Abstract

**Rationale:**

Cystic duct (CD) variations are common biliary tract alterations; therefore, caution is required in the diagnosis and treatment of cholelithiasis in patients with gallbladder duct anomalies. Laparoscopic cholecystectomy (LC) is the preferred method for treating cholelithiasis or cholecystitis. Accurate preoperative imaging assessment, a thorough understanding of relevant surgical anatomy, and intraoperative precautions are required to effectively reduce the incidence of intraoperative bile duct injury.

**Patient concerns:**

We report the case of a 59-year-old male patient with common hepatic duct (CHD) stricture following LC. The patient’s CD entered the common bile duct from the left side and ran ventrally around the common bile duct. Postoperative examination revealed partial stenosis of the CHD caused by compression from an absorbable ligation clip and transient hyperbilirubinemia. Fortunately, prompt treatment prevented serious complications.

**Diagnoses:**

Cholelithiasis with cholecystitis and CD anatomical variation.

**Interventions:**

LC.

**Outcomes:**

Postoperative examination revealed partial stenosis of the CHD caused by compression from an absorbable ligation clip, with transient hyperbilirubinemia. The patient was discharged 3 days postoperatively and followed up for 10 months. The absorbable ligation clips were resorbed, and the CHD stenosis resolved.

**Lessons:**

Biliary tract surgeons must understand and master common variations of the CD and their clinical significance. Potential anatomical variations should be identified preoperatively, while the extrahepatic bile ducts should be carefully exposed intraoperatively. Whenever possible, absorbable ligation clips should be used to transect the CD while avoiding compression of the bile duct to prevent bile duct injury.

## 1. Introduction

Cholelithiasis is a prevalent benign gallbladder disease, with an estimated incidence of 5% to 15%.^[1]^ Laparoscopic cholecystectomy (LC) is the standard procedure for treating benign gallbladder diseases, such as cholelithiasis and cholecystitis, because of its minimal invasiveness and rapid postoperative recovery.^[2,3]^ Previous reports indicate that the incidence of bile duct injury (BDI) during LC varies, with LC being one of the leading causes of iatrogenic BDI.^[4–6]^ BDI can result in serious complications, including bile leakage, biliary stricture, biliary cirrhosis, portal hypertension, and even liver failure. These complications not only compromise the patient’s safety and quality of life but also increase the risk of medical disputes. Anatomical variations of the biliary tract are one of the primary causes of BDI.^[7]^ Considering the potential severity of this complication, surgeons should endeavor to minimize the risk of BDI. For patients with suspected biliary tract variations, a comprehensive preoperative evaluation is essential. Such imaging modalities include magnetic resonance imaging (MRI), magnetic resonance cholangiopancreatography (MRCP), and contrast-enhanced computed tomography. Meticulous intraoperative techniques, combined with advanced imaging technologies, are essential to reduce iatrogenic injury. This report describes a case of common hepatic duct (CHD) stenosis after LC in a patient whose CD joined the common bile duct on the ventral aspect from the left side. Postoperatively, the patient developed partial stenosis of the CHD due to compression from an absorbable ligation clip and transient hyperbilirubinemia. Fortunately, no severe complications occurred following timely intervention. A detailed report of this case is warranted to raise awareness among biliary surgeons and help prevent similar BDIs.

## 2. Case presentation

**Figure 1.**
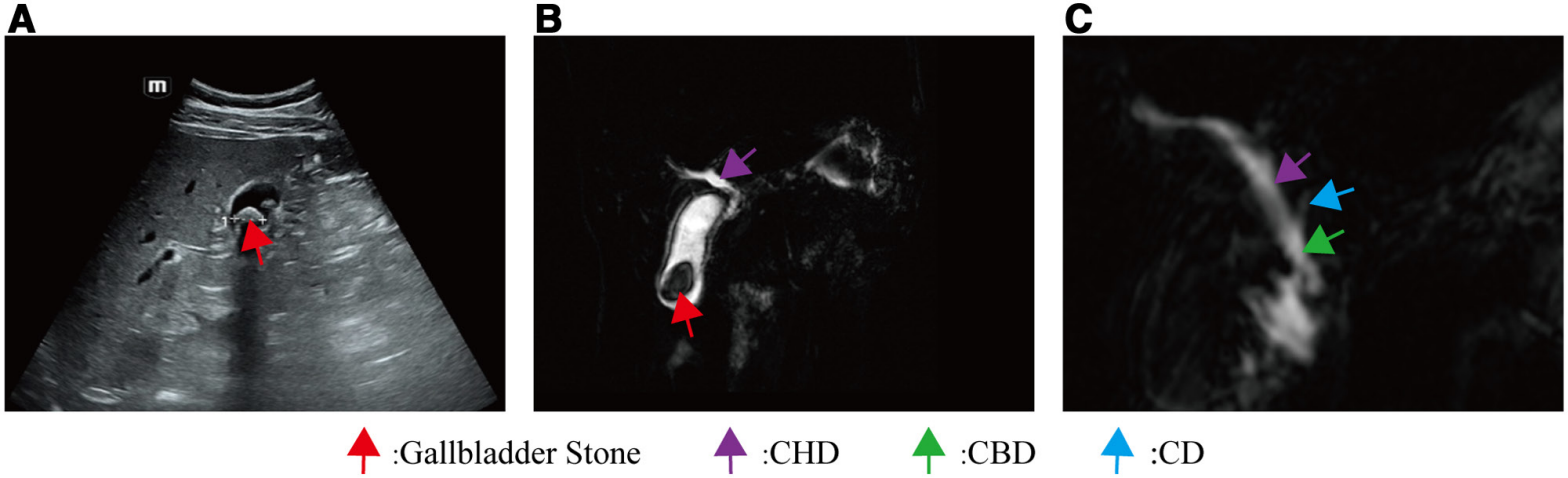
Preoperative abdominal ultrasound and MRI imaging studies. (A) Abdominal ultrasound indicates gallstones and gallbladder wall thickening. (B) MRCP confirmed signs of cholelithiasis and cholecystitis. (C) MRCP shows the cystic duct joining the common bile duct from the left side. CBD = common bile duct, CD = cystic duct, CHD = common hepatic duct, MRCP = magnetic resonance cholangiopancreatography, MRI = magnetic resonance imaging.

The patient provided written informed consent for their information to be used in this case report. The study was reviewed and approved by the Ethics Committee of Jintang Second People’s Hospital (Approval No. IEC-LW2026001). The patient was a 59-year-old male patient who was admitted to the Hepatobiliary Surgery Department of Jintang Second People’s Hospital with a 1-week history of right upper abdominal pain. The pain was described as persistent and distending, located in the right upper abdomen, radiating to the back, and associated with nausea without vomiting. There was no history of chills or fever. The patient had no prior abdominal surgery and no relevant family medical history. Vital signs, including temperature, respiratory rate, pulse rate, and blood pressure, were all within normal limits. The patient’s skin and sclera were not jaundiced. Cardiopulmonary examination revealed no abnormalities. The abdomen was generally soft, with right upper quadrant tenderness and a positive Murphy sign; however, there was no rebound tenderness or muscle rigidity. Upper abdominal ultrasound revealed gallstones and gallbladder wall thickening (Fig. 1A). Upper abdominal MRI and MRCP revealed cholelithiasis and gallbladder wall edema (Fig. 1B) and showed that the CD coursed ventrally to the CHD and joined the common bile duct from the left side (Fig. 1C). According to the “Expert Consensus on Surgical Treatment of Benign Gallbladder Diseases (2021 Edition)” by the Biliary Surgery Group of the Surgery Branch of the Chinese Medical Association, the patient had clear surgical indications and no contraindications. Based on the patient’s preference, LC was performed on December 24, 2024. Preoperative laboratory tests showed that both blood cell counts and bilirubin levels were within normal limits. Specific values are summarized in Table 1.

**Table 1:** The patient’s WBC, NEUT%, Hs-CRP, and bilirubin levels.

Variables	Preoperative	Postoperative day 1	Postoperative day 3	Postoperative day 14	Postoperative 3-month	Postoperative 10-month
WBC (4–10) (×10^9^/L)	10.09	9.33	6.99	N/A	N/A	N/A
NEUT% (50–70)	71.0	78.9	77.2	N/A	N/A	N/A
Hs-CRP (0–10) (mg/L)	0.75	1.05	16.3	N/A	N/A	N/A
TBIL (5.1–19) (µmol/L)	18.7	49.4	23.7	16.9	16.3	15.5
DBIL (1.7–6.8) (µmol/L)	9.2	31.6	9.4	6.7	6.2	6.5
IBIL (0–18) (µmol/L)	9.5	17.8	14.3	10.2	10.1	9.0

DBIL = direct bilirubin, Hs-CRP = high-sensitivity C-reactive protein, IBIL = indirect bilirubin, N/A = no data, NEUT% = neutrophil percentage (%), TBIL = total bilirubin, WBC = white blood cells.

**Figure 2.**
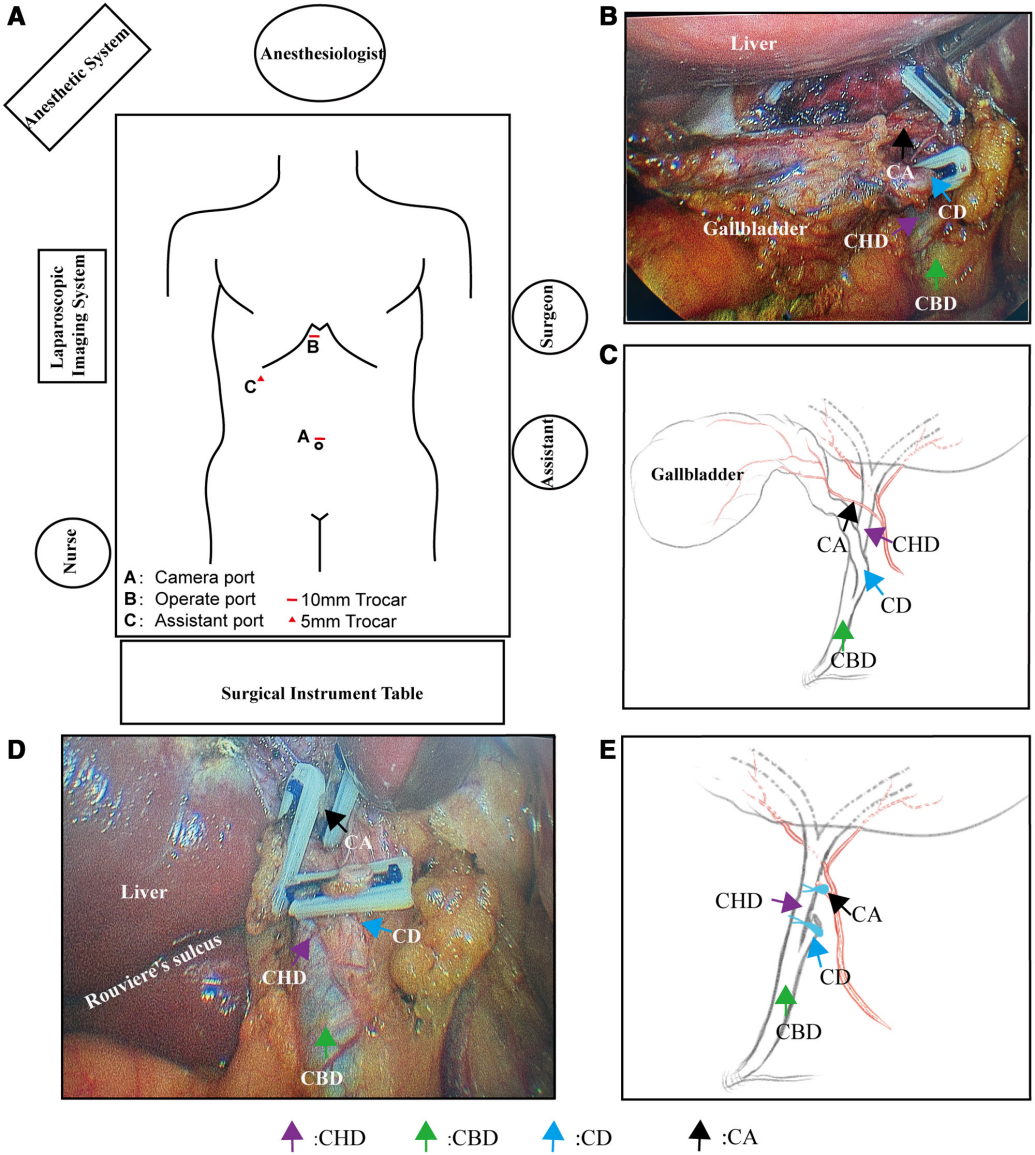
Schematic diagram of the surgical layout and intraoperative anatomical variations. (A) Schematic diagram of surgical personnel, equipment positioning, and trocar layout. (B) The cystic duct and cystic artery pass anterior to the common hepatic duct and join from the left side. (C) Schematic diagram of cystic duct variations observed intraoperatively. (D) The position of the bile duct and absorbable ligation clips after cholecystectomy. (E) Schematic diagram of cystic duct variations and absorbable ligation clip positions after cholecystectomy. CA = cystic artery, CBD = common bile duct, CD = cystic duct, CHD = common hepatic duct.

**Figure 3.**
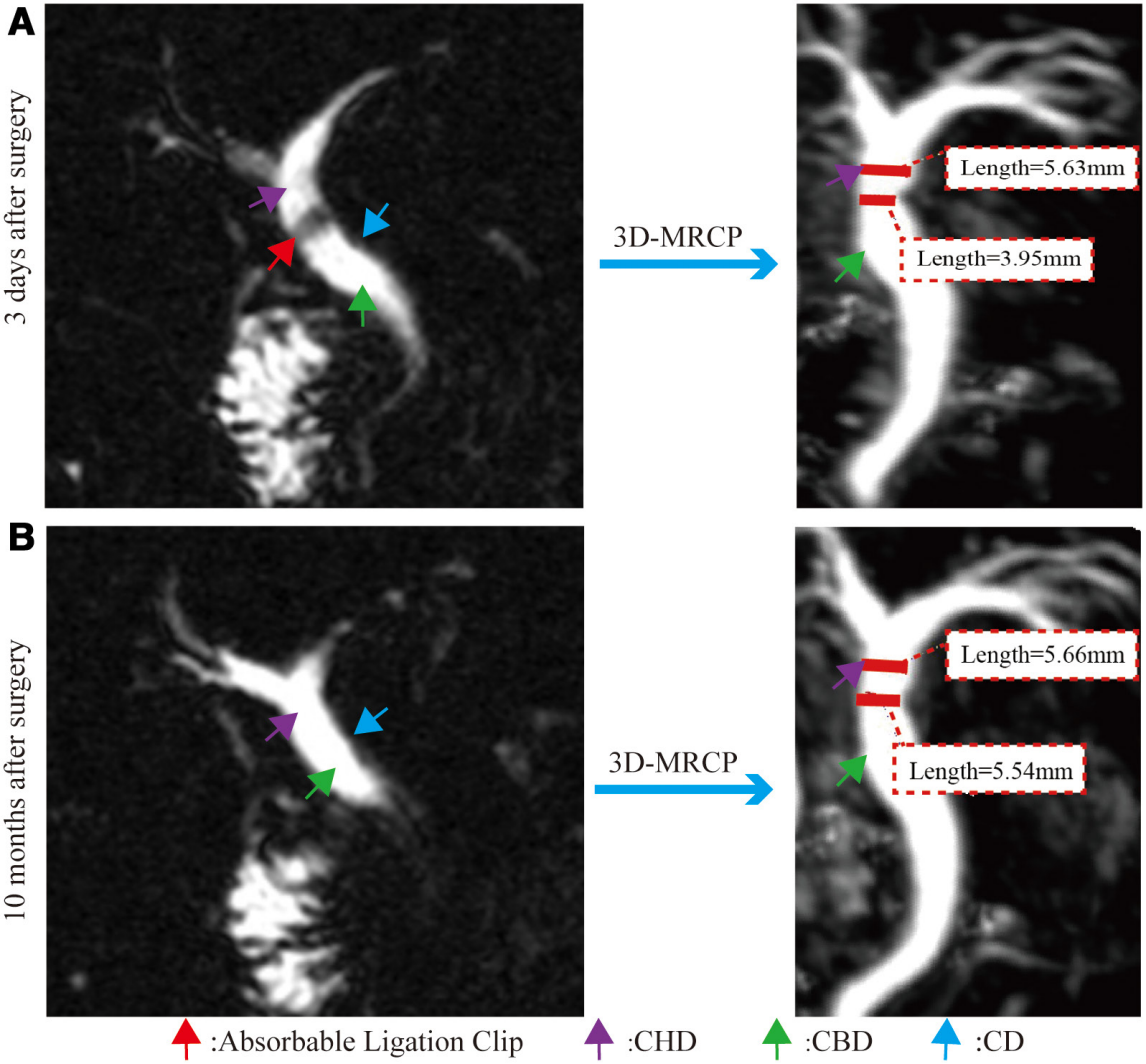
MRCP examination at 3 days and at 10 months postoperatively. (A) MRCP reexamination 3 days postoperatively indicated partial stenosis of the CHD due to compression by absorbable clips. (B) MRCP reexamination 10 months postoperatively shows that the absorbable clip had been completely absorbed, and the incomplete stenosis of CHD has essentially disappeared with a patent CHD. CA = cystic artery, CBD = common bile duct, CD = cystic duct, CHD = common hepatic duct, MRCP = magnetic resonance cholangiopancreatography.

The patient underwent conventional 3-port LC. The surgical team positions and laparoscopic setup are shown in Figure 2A. Following induction of anesthesia and standard preparation, a 10-mm supraumbilical incision was made. Pneumoperitoneum was established using the Hasson technique at 14 mm Hg. A laparoscope was inserted, and under direct vision, a 10-mm trocar and a 5-mm trocar were placed 1 cm below the xiphoid process and right subcostal margin, respectively (Fig. 2A). The surgeon retracted the gallbladder neck using the left-hand instrument via the right upper abdominal trocar, exposing the anterior and posterior peritoneal layers of the Calot triangle. Dissection with an electrocautery hook through the subxiphoid trocar allowed meticulous isolation of the cystic artery and CD. Intraoperative confirmation revealed that the CD coursed ventrally to the CHD and joined the left side of the common bile duct (Fig. 2B). Figure 2C shows a schematic diagram of the variant anatomy. The CD was clamped with an absorbable ligating clip (Zhejiang Shengshi Technology Co., Ltd., China) 0.5 cm from its junction with the common bile duct without transection. The gallbladder was then dissected retrogradely from the liver. After confirming the CD and artery within the Calot triangle, both structures were transected distal to the applied absorbable ligating clip (Fig. 2D). Figure 2E shows the anatomy after gallbladder removal and clip placement. After securing hemostasis and confirming the absence of bile leakage, the gallbladder and stones were removed via the subxiphoid incision. The pneumoperitoneum was then released, and all incisions were closed. Gross examination of the gallbladder confirmed gallstones, and histopathology confirmed cholecystitis. On postoperative day 1, the patient developed a fever with a temperature of up to 38.5°C. A repeat complete blood count revealed normal findings; however, liver function tests showed elevated bilirubin levels compared with preoperative levels (Table 1). MRI/MRCP revealed partial stenosis of the CHD caused by compression from the absorbable ligating clip (Fig. 3A). Specifically, the diameter of the CHD at the narrowed segment was approximately 3.95 mm, compared with approximately 5.63 mm in the proximal segment, corresponding to an estimated luminal narrowing of approximately 30% (Fig. 3A). The patient received physical cooling and oral ademetionine 1,4-butanedisulfonate 500 mg twice daily, after which his body temperature normalized. On postoperative day 3, bilirubin levels had returned to normal, and the patient was discharged without discomfort. Two-week follow-up liver function tests showed normal bilirubin levels. Follow-up at 3 and 10 months showed normal bilirubin levels. Specific values are summarized in Table 1. MRI/MRCP demonstrated complete absorption of the ligating clip, resolution of the partial CHD stenosis, and patency of the duct (Fig. 3B).

## 3. Discussion

Although LC is widely performed and generally considered safe, intraoperative and postoperative complications still occasionally occur, particularly iatrogenic injuries arising from anatomical variations of the bile ducts. Proper ligation of the CD and artery is a crucial surgical step. Inadequate ligation can result in bile leakage, hemorrhage, conversion to open surgery, and long-term complications, including bile duct strictures or recurrent bile duct stones.^[8]^ Traditional metal clips, such as titanium clips, have long been the preferred method for duct closure owing to their ease of use, widespread availability, and low cost. However, complications related to metal clips have increasingly attracted attention, including clip migration causing bile duct stones, cholangitis, bile duct strictures, and incomplete occlusion of the CD.^[9–11]^ To reduce these risks, polymer-based closure clips, including both absorbable and nonabsorbable types, have been gradually adopted in clinical practice. Their advantages include versatility, structural stability, ease of use, and noninterference with imaging studies.^[12]^ However, this case report presents a partial stricture of the CHD caused by compression from an absorbable ligating clip after LC, emphasizing the potential complications associated with this seemingly safe device. Compared with previous reports, short-term bile duct strictures caused by clamp compression after LC are rare; most documented cases involve long-term displacement of nonabsorbable ligation clamps.^[13–15]^ The mechanism of clip migration remains incompletely understood. Based on the literature and the features of this case, we propose that its occurrence results from the combined influence of multiple factors. First, technical factors should be considered. If the clamping site is too close to the common bile duct or if the CD stump is too short, excessive traction during clamping may inadvertently involve surrounding tissues or even a small segment of the common bile duct wall. Excessive or overlapping clamping of tissue may result in bile duct stenosis. In this case, compression and stricture of the CHD were observed as early as 3 days postoperatively. This was likely due to the CD originating from the left side, which caused the stump to retract and compress the CHD. Second, local inflammatory factors may contribute significantly. The patient had preoperative cholecystitis and an underlying peribiliary infection, which was further aggravated by postoperative inflammation and edema.^[16]^ Finally, intraoperative gallbladder rupture with bile leakage may provoke delayed local inflammation. This may result in detachment of ligature clamps on the CD or artery due to tissue necrosis or corrosion, potentially causing clamp displacement or even migration into the bile duct.^[15,17]^

To minimize the risk of the rare complication of bile duct stenosis caused by tissue displacement, preventive strategies should be applied across all stages of care: preoperative, intraoperative, and postoperative. First, the course of the bile ducts and their anatomical variations should be carefully evaluated before surgery. Surgeons should be well acquainted with bile duct variants and identify high-risk patients.^[18]^ Second, surgeons should accurately identify the structures within the Calot triangle during surgery, maintain a loose clamp when handling the CD, and avoid excessive traction at its junction with the bile duct. The CD stump should be at least 0.5 cm long to prevent the ligation clamp from compressing the common bile duct.^[19]^ In addition, energy-based devices, such as the ultrasonic scalpel, can precisely sever tissue, reducing the number of clips needed and minimizing the risk of displacement.^[20]^ In terms of closure material selection, absorbable clips, owing to their excellent biocompatibility and in vivo degradability, can reduce the risk of long-term bile duct strictures.^[8,21]^ The absorbable ligating clip used in this case features a polyglactide outer layer and a polydioxanone inner layer, and is fully absorbed within approximately 39 weeks. In this case, the absorbable ligating clip may have mitigated the risk of long-term bile duct stricture due to its biological properties, although long-term follow-up is still required. Nonabsorbable sutures, however, may provide better cost-effectiveness regarding procedure time and expense; the choice should be individualized based on the patient’s condition, intraoperative findings, inflammation severity, and hospital resources. In addition, suture ligation remains an economical and reliable method for closing dilated or edematous CDs.^[22]^ Finally, postoperative follow-up and management are critical. Vital signs and liver function should be closely monitored, and regular imaging, such as MRI or ultrasound, should be conducted. Patients should be advised to avoid strenuous activity, facilitating early detection and timely intervention for any abnormalities.

This study has several limitations. First, as a single-case report, the generalizability of the findings is inherently limited. The true incidence, risk factors, and optimal prevention and management strategies for absorbable clip displacement remain to be established through future studies with larger sample sizes. Nevertheless, this case provides a comprehensive account of the entire process, from preoperative evaluation to intraoperative procedures and postoperative follow-up, offering a valuable reference for clinicians in preventing and managing similar rare complications.

## 4. Conclusions

Stenosis of the CHD due to compression from absorbable ligation clips is a rare but clinically significant complication following LC. Even with adherence to protocols, surgeons must be vigilant about the spatial relationship between ligating instruments and the bile duct. Accurate preoperative assessment, standardized intraoperative techniques, careful selection of closure devices, and systematic postoperative follow-up are essential for preventing iatrogenic BDI. Future large-scale, multicenter studies could further clarify risk factors and optimize diagnostic and therapeutic strategies, which may ultimately reduce the incidence of biliary complications.

## Author contributions

**Data curation:** Kun He, Wei Cui.

**Funding acquisition:** Kun He.

**Visualization:** Kun He, Xiaojin Gao.

**Methodology:** Houzu Zhou.

**Writing – original draft:** Kun He, Xiaojin Gao.

**Writing – review & editing:** Mengyi Xie, Yao Liu, Jingdong Li.
